# Dynamic changes in the yak rumen eukaryotic community and metabolome characteristics in response to feed type

**DOI:** 10.3389/fvets.2022.1027967

**Published:** 2022-12-22

**Authors:** Xiaojing Cui, Yue Liu, Hao Wu, Qingxiang Meng, Shujie Liu, Shatuo Chai, Lizhuang Hao, Zhenming Zhou

**Affiliations:** ^1^State Key Laboratory of Animal Nutrition, College of Animal Science and Technology, China Agricultural University, Beijing, China; ^2^Qinghai Academy of Animal and Veterinary Sciences, Qinghai University, Xining, China

**Keywords:** yak, rumen, microbiota, fungi, protozoa, metabolomics, feed type

## Abstract

With diversification of yak breeding, it is important to understand the effects of feed type on the rumen, especially microbiota and metabolites. Due to the unique characteristics of yak, research on rumen microbes and metabolites is limited. In this study, the effects of two diet types on rumen eukaryotic microflora and metabolites were evaluated using the Illumina MiSeq platform and liquid chromatography-mass spectrometry (LC-MS). All identified protozoa belonged to *Trichostomatia*. At the genus level, the relative abundance of *Metadinium* and *Eudiplodinium* were significantly (*p* < 0.05) higher in the roughage group than that of concentrate group, while the concentrate group harbored more *Isotricha*. *Ascomycota, Basidiomycota*, and *Neocallimastigomycota* were the main fungal phyla, and the *Wallemia, Chordomyces, Chrysosporium, Cladosporium, Scopulariopsis*, and *Acremonium* genera were significantly (*p* < 0.05) more abundant in the roughage group than the concentrate group, while the concentrate group harbored more *Aspergillus, Neocallimastix, Thermoascus*, and *Cystofilobasidium* (*p* < 0.05). Metabolomics analysis showed that feed type significantly affected the metabolites of rumen protein digestion and absorption (L-proline, L-phenylalanine, L-tryosine, L-leucine, L-tryptophan, and β-alanine), purine metabolism (hypoxanthine, xanthine, guanine, guanosine, adenosine, and adenine), and other metabolic pathway. Correlation analysis revealed extensive associations between differential microorganisms and important metabolites. The results provide a basis for comprehensively understanding the effects of feed types on rumen microorganisms and metabolites of yaks. The findings also provide a reference and new directions for future research.

## 1. Introduction

Ruminant and rumen microbes are closely dependent on each other. These bacteria, make up 90% of the microbial population, and eukaryotes (fungi and protozoa), also make an important though poorly understood contribution (1–3). Fungi play pivotal role in the degradation of dietary fiber (4, 5), and protozoa function in carbohydrate degradation and maintaining rumen pH (6). Changes in the host environment affect rumen microbes, among which diet is the main factor affecting the microbial community (7). Many previous studies analyzed microbial and metabolite changes to comprehensively explore the effects of diet type on the rumen and ruminants. The findings suggest that dietary changes can alter the rumen microbiota, modifying rumen metabolites. Metabolites can reflect microbial interactions with diet, and this can impact the health and performance of ruminants (8–10).

Yak is an important ruminant animal in the plateau area of China, accounting for >90% of worldwide yak production (11, 12). When a traditional grazing pattern is employed, yak weight varies seasonally, which affects animal productivity and the income of herdsmen (13, 14). Nowadays, two modes are common; two-stage feeding (grazing in the warm season, shelter feeding in the cold season) and shelter fattening (12, 15). Supplementary feeding with total mixed rations of available roughage and grains is not entirely dependent on grazing (16). However, yak has evolved a unique rumen microflora and metabolic mechanism adapted to the harsh environment (17–20). Little information is available on the effects of different feed type on yaks to make appropriate supplementary feeding strategy.

Therefore, we explored the effects of feed type on the rumen bacterial community and metabolism in yak in our previous work (21). However, our knowledge of eukaryotic communities and metabolites in yak remains limited. Only through investigating the microflora and metabolites will we be able to better explain the effects of feed type on yak rumen. Herein, we combined microbial sequencing and metabolomics techniques to explore the effects of two different feed types on rumen eukaryotes microorganisms and metabolites, and the possible relationships between ruminal microbiota and metabolites.

## 2. Materials and methods

### 2.1. Animals, treatments, and feeding regimes

The study was conducted at Haibei Tibetan Autonomous Prefecture, Qinghai Province of China. This area is over 3,000 m above sea level and the annual average temperature is 1.5°C. Sixteen male Datong yaks aged 48 months and initial BW (195 ± 50 kg) resided at Haibei Tibetan Autonomous Prefecture Plateau Ecological Animal Husbandry science and technology Demonstration park. These animals were used as experimental animals and randomly divided into four groups. Four 4 × 4 Latin square experiments were conducted, with four diets per square, making 16 different diets. Each experiment lasted 21 days, and the adaptation period lasted 15 days. According to the fiber content, feed with crude fiber content below 18% was placed in the concentrate feed group (CFG), and feed with crude fiber content of 25–45% was placed in the roughage feed group (RFG). The concentrate feed group included soybean meal, broad bean, rapeseed meal, sesame meal, oat, hulled barley, corn, barley, wheat, and wheat bran. The roughage feed group consisted of wheat straw, pea stem, broad bean stem, rape straw, oat straw, and alfalfa. Feed in the roughage feed group was fed separately, and feed in the concentrate feed group was processed into pellets using a diameter of 6 mm with 30% oat straw. Diets were supplemented with 2% buffering additives (NaHCO_3_ and MgO with 2:1 ratio), 0.5% limestone, 0.5% NaCl, and 30 mg/kg rumensin to maintain normal rumen fermentation. Yaks were fed 1.9% bodyweight (BW) on a dry matter basis at 0800 and 1,600. Experiments were performed in accordance with the Regulations for the Administration of Affairs Concerning Experimental Animals (The State Science and Technology Commission of P. R. China, 1988). The procedures for the care and use of animals in this study were approved and conducted according to standards established by the College of Animal Science and Technology, CAU, Beijing, P. R. China (permit number DK1402006).

### 2.2. Rumen sampling and measurements

Rumen fluid was collected from yaks before feeding on the morning of day 22. A 50 mL syringe was used to extract rumen fluid from the oral cannula. The collecting device was thoroughly washed with clean warm water and the first rumen fluid sample collected was discarded. About 50 mL of rumen fluid was then collected from each animal and transferred to a 50 mL cryopreservation tube and frozen in liquid nitrogen. A total of 64 rumen fluid samples were collected from yaks, but some samples were damaged due to improper storage, and 40 samples were used for follow-up analysis.

### 2.3. DNA extraction, PCR amplification, and high-throughput sequencing

Microbial community genomic DNA was extracted from rumen samples using an E.Z.N.A. SOIL DNA Kit (Omega Bio-Tek, Norcross, GA, U.S.) according to the manufacturer's instructions. Extract DNA was checked on a 1% agarose gel, and the concentration and purity were determined using a NanoDrop 2000 UV-vis spectrophotometer (Thermo Scientific). Protozoa primers reg1320R (5′-AATTGCAAAGATCTATCCC-3′) and RP841F (5′-GACTAGGGATTGGARTGG-3′) (7) and fungal primers ITS1F (5′-CTTGGTCATTTAGAGGAAGTAA-3′) and ITS2R (5′-GCTGCGTTCTTCATCGATGC-3′) (22) were used for PCR amplification of variable regions. PCR mixtures contained 4 μL of 5× TransStart FastPfu buffer, 2 μL of 2.5 mM dNTPs, 0.8 μL of forward primer (5 μM), 0.8 μL of reverse primer (5 μM), 0.4 μL of TransStart FastPfu DNA Polymerase, 10 ng of template DNA, and double-distilled water (ddH_2_O) up to 20 μL. Amplifications were performed in triplicate. PCR products were extracted from 2% agarose gels and purified using an AxyPrep DNA Gel Extraction Kit (Axygen Biosciences, Union City, CA, USA) according to the manufacturer's instructions, and quantified using a Quantus™ Fluorimeter (Promega, USA). Purified amplicons were pooled in equimolar amounts and paired-end (PE) sequenced (2 × 300) on an Illumina MiSeq platform (Illumina, San Diego, CA, USA) according to standard protocols by Majorbio Bio-Pharm Technology Co. Ltd. (Shanghai, China).

### 2.4. Data analysis

Raw sequencing reads were demultiplexed and quality-filtered by Fastp (version 0.19.6, https://github.com/OpenGene/fastp) and merged by FLASH (version 1.2.11, https://ccb.jhu.edu/software/FLASH/index.shtml) with the following criteria: (i) 300 bp reads were truncated at any site receiving an average quality score of <20 over a 50 bp sliding window, truncated reads shorter than 50 bp were discarded, and reads containing ambiguous bases were also discarded; (ii) according to overlap relation between PE reads, pairs were merged into sequences with a minimum length overlap of 10 bp; (iii) the maximum mismatch ratio of overlap regions was 0.2. Reads that could not be assembled were discarded. Operational taxonomic units (OTUs) with a 97% similarity cutoff were clustered using UPARSE (version 11, http://drive5.com/uparse/), and chimeric sequences were identified and removed. The taxonomy of each OTU representative sequence was analyzed by RDP Classifier (version 2.13, https://sourceforge.net/projects/rdp-classifier/) against the 18S rRNA database (Protist_PR2_v4.5) and the ITS database (unite 8.0) using a confidence threshold of 0.7.

The analysis was conducted using the free online Majorbio I-Sanger Cloud Platform (http://www.i-sanger.com). The alpha-diversity index was calculated using MOTHUR (version v.1.30.2, https://www.mothur.org/wiki/Download_mothur). The R language tool was used to generate dilution curves and plot bar charts related to results. Beta-diversity was estimated by calculating the unweighted UniFrac distance and visualized using principal coordinate analysis (PCoA), and the results were plotted using GUniFrac and ape packages in R (23, 24). The Wilcoxon rank-sum test within STAMP (version v.2.1.3) was used to identify phyla and genera that showed significant differences in abundance between groups (confidence interval method) (25). Spearman's correlation analysis was used to evaluate correlations between different metabolites and microbial communities using the pheatmap software package in R (version 61, http://CRAN.R-project.org/package=pheatmap), with *p* < 0.05 indicating statistical significance.

## 3. Results

### 3.1. Diversity of the rumen microbiota of yaks receiving different feed types

**Figure 1:**
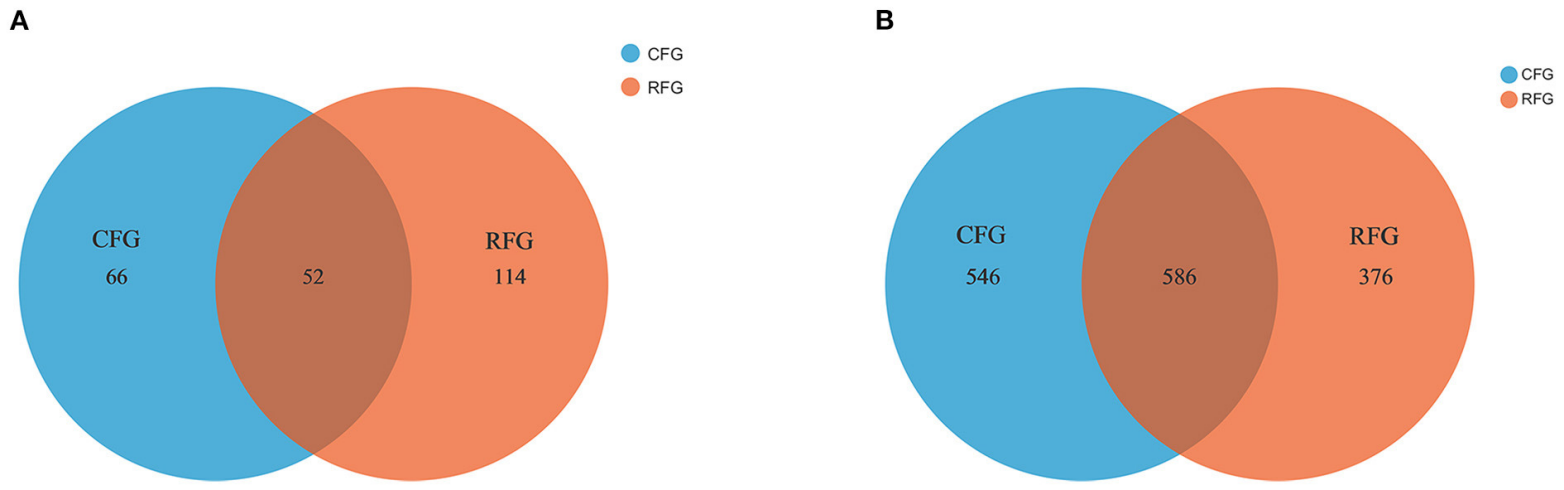
Protozoa **(A)** and fungi **(B)** identified in the yak rumen. Shared OTUs across different groups are indicated. CFG, concentrate feed group; RFG, roughage feed group.

**Figure 2:**
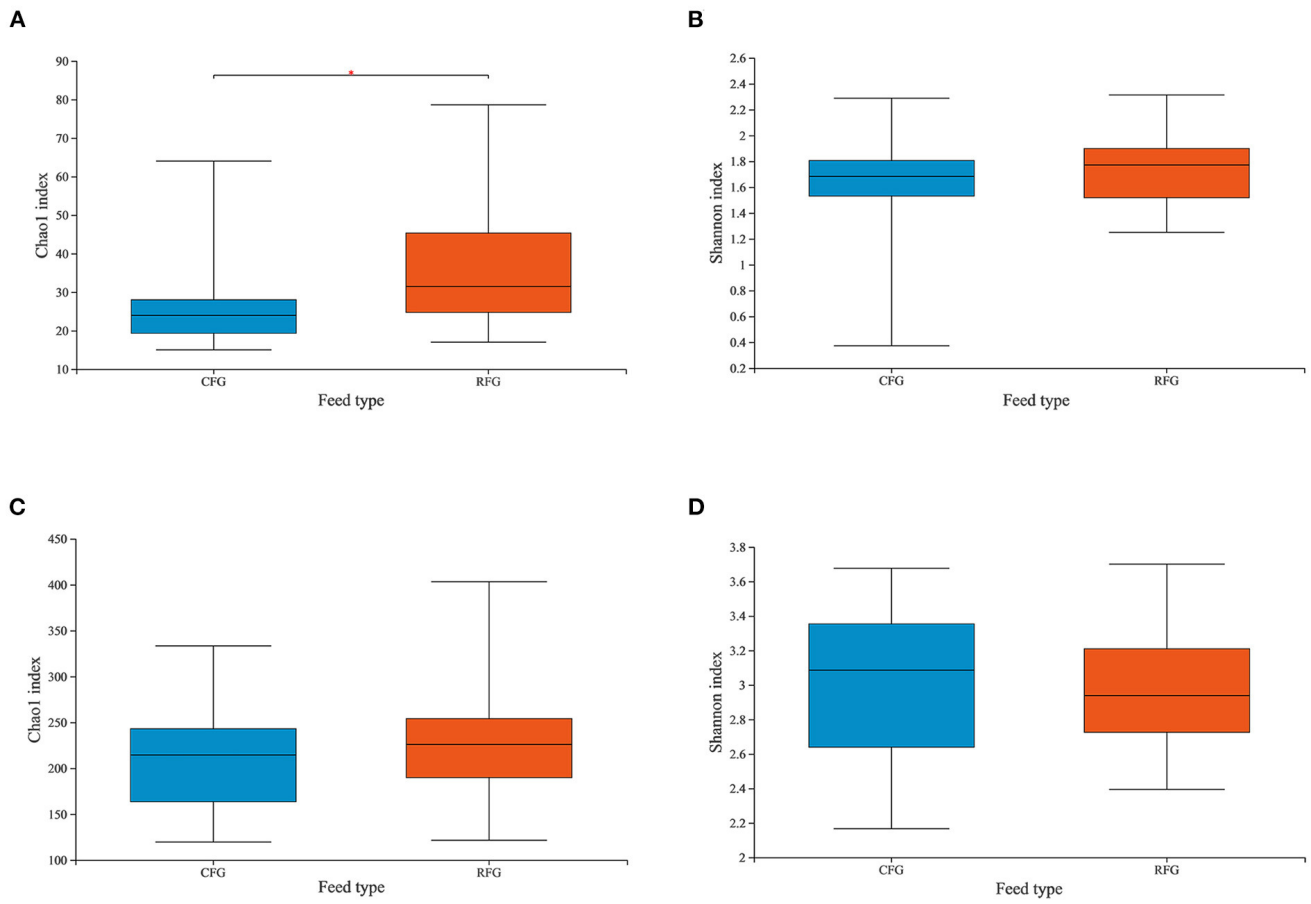
Differences in Yak ruminal protozoal **(A, B)** and fungal **(C, D)** diversity and richness between the two groups. Microbial richness was estimated by Chao1 value. Microbial diversity was estimated by Shannon index; *indicates significant differences between the two group (*p* < 0.05).

In this study, high-quality 1,771,085 protozoal sequences and 2,245,482 fungal sequences were obtained. Each sample sequence was processed according to a minimum number of samples. Venn diagrams revealed the number of core and differing microbes (Figure 1). For protozoa, the total number of OTUs in CFG and RFG was 118 and 166, respectively, and 52 shared OTUs. For fungi, a total of 546 and 376 OTUs were unique to the CFG and RFG, and 586 shared OTUs. The protozoal community richness (Chao 1) for RFG was greater than that for CFG (*p* = 0.049; Figure 2). Community diversity (Shannon) of fungi and protozoa showed no significant differences between the two groups.

### 3.2. Microbial communities of the yak rumen microbiota following different feed types

**Figure 3:**
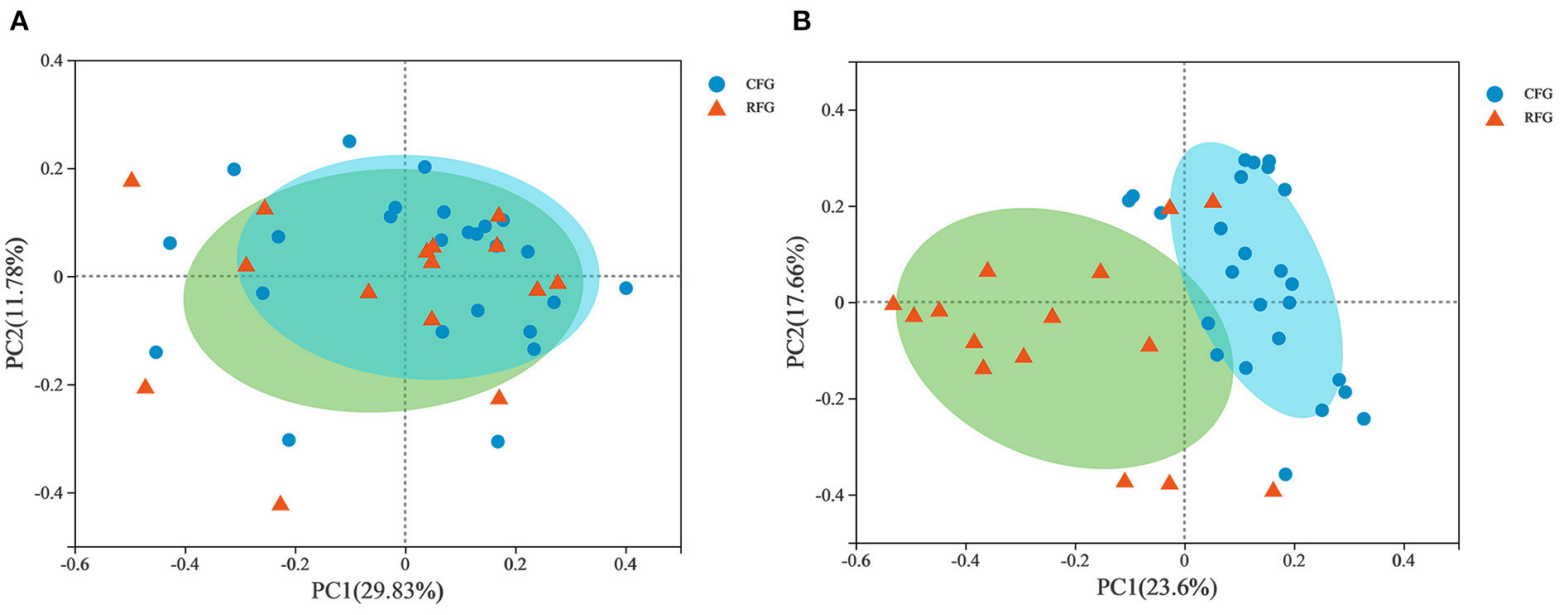
Principal coordinate analysis (PCoA) of rumen protozoal **(A)** and fungal **(B)** communities.

In Figure 3, PCoA plot clearly showed that fungal samples were not completely separated by feed type, but protozoal samples revealed aggregation. This results indicated that there were some differences in fungal community structure between CFG and RFG, while the protozoal community structure was similar.

**Figure 4:**
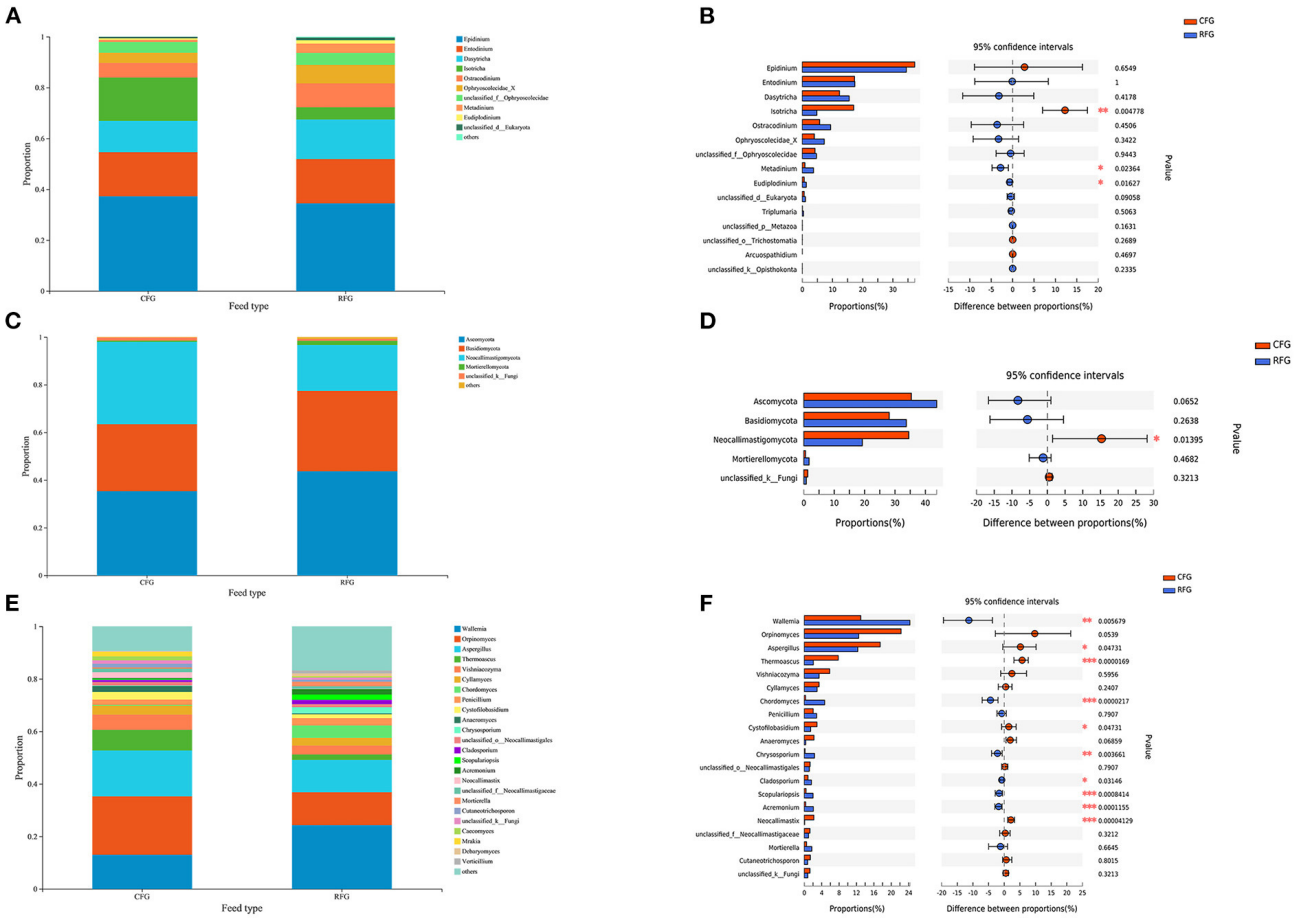
Classification of protozoal and fungal community composition in the two groups. **(A)** Protozoa at the genus level. **(B)** Extended error bar plot showing protozoa at the genus level that differ significantly between the two groups. **(C)** Fungi at the phylum level. **(D)** Extended error bar plot showing fungi at the phylum level that differ significantly between the two groups. **(E)** Fungi at the genus level. **(F)** Extended error bar plot showing fungi at the genus level that differ significantly differences between the two groups; *0.01 < *p* ≤ 0.05; **0.001 < *p* ≤ 0.01; ***p ≤ 0.001.

As shown in Figure 4, all protozoa indentified in this study belonged to *Trichostomatia*, and they could be divided into *Ophryoscolecidae* and *Isotrichidae* at the family level. The fungal phyla *Ascomycota* (43.68%), *Basidiomycota* (33.71%), and *Neocallimastigomycota* (19.25%) were the most abundant. The relative abundance of *Neocallimastigomycota* was significantly higher CFG than in RFG (*p* = 0.014).

For protozoa, *Epidinium* was the most abundant genus, accounting for ~35%, far surpassing the second and third most abundant protozoa. The relative abundance of *Isotricha* in CFG was significantly higher than in RFG (*p* = 0.005). By contrast, the relative abundance of *Metadinium* and *Eudiplodinium* in RFG was significantly higher than in CFG (*p* < 0.05). At the genus level, *Orpinomyces, Wallemia*, and *Aspergillus* were the dominant genera for fungi, collectively accounting for ~50% of fungal. The relative abundances of *Wallemia, Chordomyces, Chrysosporium, Cladosporium, Scopulariopsis*, and *Acremonium* were higher in RFG than CFG (*p* < 0.05). However, the relative abundances of *Aspergillus, Thermoascus, Cystofilobasidium*, and *Neocallimastix* were significantly higher in CFG than RFG (*p* < 0.05).

### 3.3. Correlations between the rumen microbiome and the metabolome

**Figure 5:**
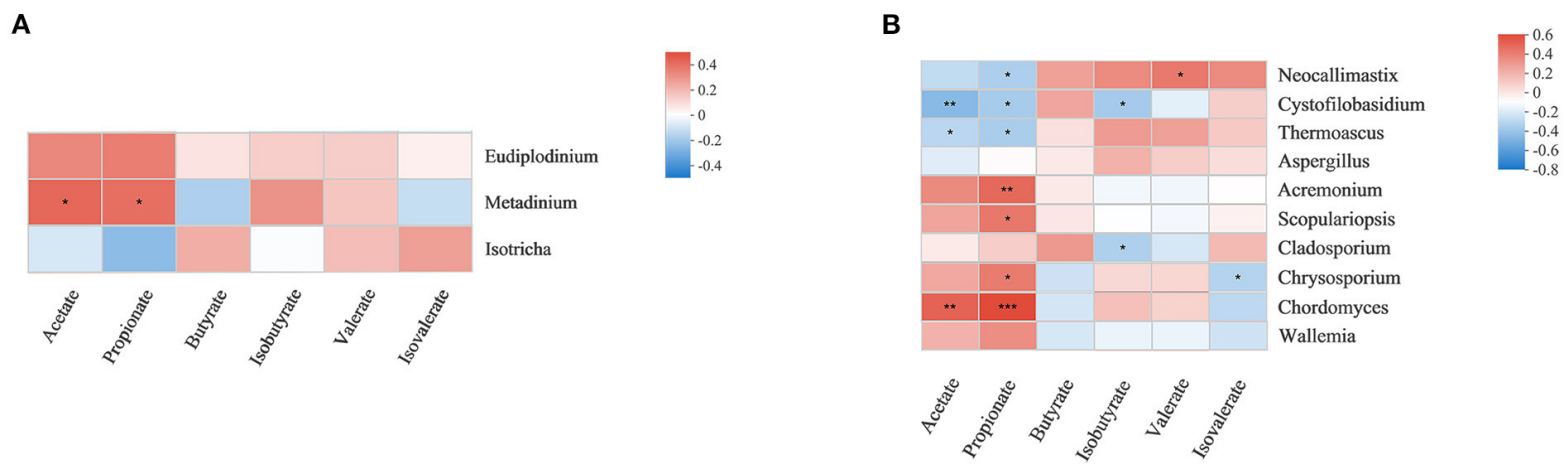
Correlations between rumen protozoa **(A)** and fungi **(B)**, and rumen fermentation parameters. Each row in the graph represents a genus, each column represents a metabolite, and each lattice represents a Spearman correlation coefficient between a component and a metabolite. Red represents a positive correlation, while blue represents a negative correlation; *0.01 < *p* ≤ 0.05; **0.001 < *p* ≤ 0.01; ***p ≤ 0.001.

Fermentation parameters and metabolomics results have been reported in previous laboratory studies (21). Herein, correlations between the relative abundances of rumen fungi and protozoa and the concentrations of metabolites were investigated by correlation analysis (Figure 5). *Metadinium* showed a significant positive correlation with acetate and propionate concentrations, and there was a significant correlation between rumen fungi and VFAs. *Chordomyces* was significantly positively correlated with acetate concentration, while *Cystofilobasidium* and *Thermoascus* were significantly negatively correlated with acetate concentration. *Chordomyces, Scopulariopsis, Acremonium*, and *Chrysosporium* were positively correlated with propionate concentration. *Neocallimastix, Cystofilobasidium*, and *Thermoascus* were significantly negatively correlated with propionate concentration. In addition, *Cystofilobasidium* and *Cladosporium* were positively correlated with isobutyrate concentration. *Neocallimastix* was significantly positively correlated with valerate concentration, while *Chrysosporium* was significantly negatively correlated with isovalerate concentration.

**Figure 6:**
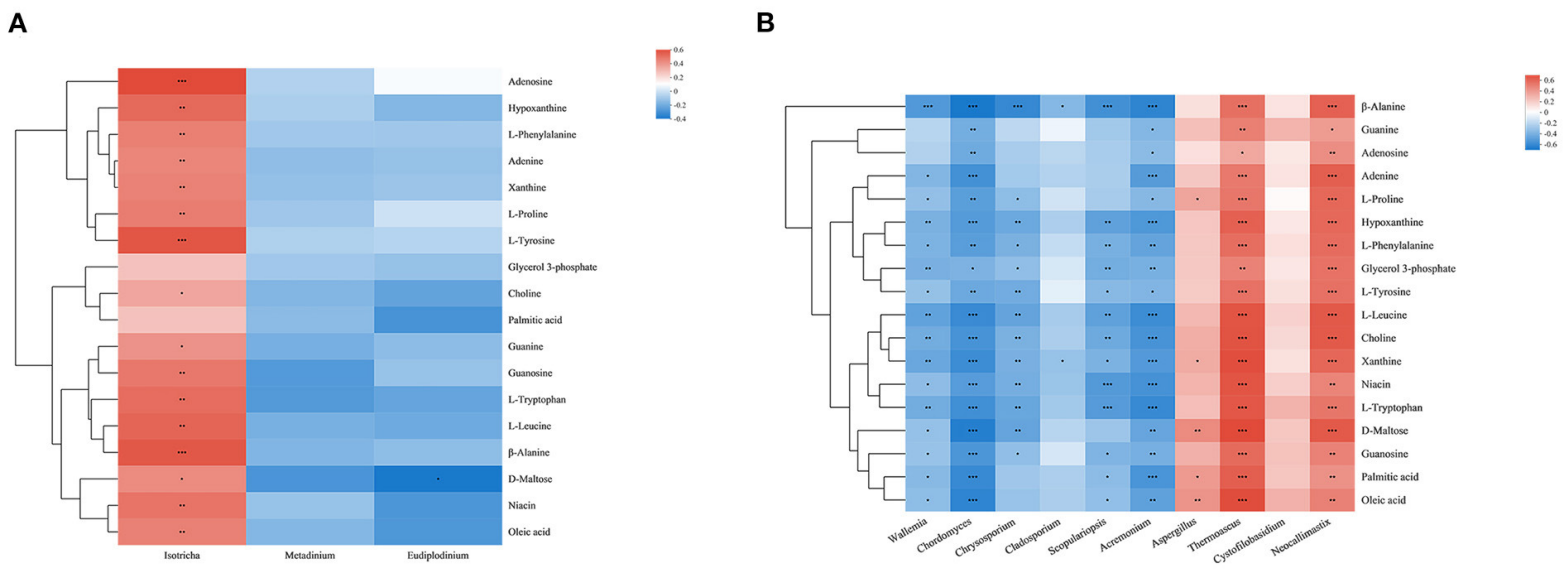
Correlation analysis between genera and metabolite concentrations affected by feed type for protozoa **(A)** and fungi **(B)**. Each row in the graph represents a metabolite, each column represents a genus, and each lattice represents a Spearman correlation coefficient between a component and a metabolite. Red represents a positive correlation, while blue represents a negative correlation; *0.01 < *p* ≤ 0.05; **0.001 < *p* ≤ 0.01; ***p ≤ 0.001.

Combined with the results of previous studies, we identified a set of metabolites that are significantly enriched in several important pathways. Metabolites were mainly divided into amino acids (L-proline, L-phenylalanine, L-tryosine, L-leucine, L-tryptophan, and β-alanine), nucleosides, purines and purine derivatives (hypoxanthine, xanthine, guanine, guanosine, adenosine, and adenine), fatty acids (palmitic acid, oleic acid), and other metabolites (choline, niacin, D-maltose, glycerol-3-phosphate). In order to explore the composition and functions of microbial communities, Spearman correlation analysis was performed on significantly different species of eukaryotic microbes at the genus level and metabolites (Figure 6). Except glycerol 3-phosphate and palmitic acid, *Isotricha* of protozoa showed a significant positive correlation with other metabolites, while fungi showed great correlations with metabolites, among which *Chordomyces, Acremonium, Thermoascus*, and *Neocallimastix* showed significant negative or positive correlation with all metabolites. *Wallemia* showed significant negative correlations with metabolites except for guanine and adenosine. *Chrysosporium* showed significant negative correlations with amino acids and some nucleosides and purine metabolites (guanosine, hypoxanthine, xanthine). *Cladosporium* showed significant negative correlations with β-alanine and xanthine. *Scopulariopsis* showed significant negative correlations with metabolites except guanine, adenosine, adenine, L-proline and D-maltose. *Aspergillus* showed significant positive correlations with L-proline, xanthine, D-maltose and fatty acids.

## 4. Discussion

Ruminants and microorganisms are in a constant dynamic balance of mutual dependence and restriction (12). The external environment can vary dramatically, and microbes change as a result, helping hosts to adapt (26). In the present work, protozoa richness changed due to dietary fiber content, but overall diversity did not change. Many studies showed that diet can affect rumen protozoa, and low concentrate diets were more conducive to the establishment of protozoa populations (27, 28). In addition, the results showed that fiber content did not change the community structure of rumen protozoa in yak, or the dominant genera. It was possible that rumen protozoa following a roughage diet were present at low density but richness is high. For fungi, changing the feed type had no effect on alpha-diversity. Consistently, previous studies also showed that dietary changes had less impact on fungal diversity (9, 10). Nevertheless, the composition of fungi varied with the type of diet. Each fungus may perform a distinct physiological function and degrade more than one substrate, which made them able to adapt to a changing diet, building resilience and resistance to change (1).

The community composition of yak protozoa was relatively simple. The rumen of most herbivorous mammals was dominated by ciliates of the subclass *Trichostomatia* (29). In this study, *Epidinium* was the dominant genus, but *Entodinium* was the most prevalent in most previous studies (9, 10, 26). *Epidinium* accounted for a large proportion of microbes in the rumen of grazing animals (27), and members of this genus secreted cellulase and hemicellulose enzymes to destroy plant cell walls and absorb chloroplasts from plant cells during fiber fermentation (30–32). It was possible that yaks evolved to preferentially host protozoa that degrade fiber to allow them to adapt to the plateau environment. Additionally, the results showed that consuming a high concentrate diet may be more favorable to *Isotricha*. It was also reported that *Isotrichidae* can rapidly use soluble sugars such as glucose, fructose, and sucrose and convert them into glycogen for storage (33). Besides, *Ophryoscolecidae* was a functionally rich population, and the results indicated that *Metadinium* and *Eudiplodinium* belonging to this order may ingest and degrade plant fibers.

The study investigated fungal community composition and abundance using the internal transcribed spacer (ITS) region, a sequence widely used in such previous studies. Anaerobic fungi, classified as phylum *Neocallimastigomycota*, were crucial in the degradation of lignocellulosic plant fiber in the rumen (4, 5, 34, 35), and they were highly abundant in CFG in the present study. This may be the result of fungi adjusting their community and mechanism to adapt to a change in diet type. In previous work, 11 genera of anaerobic fungi were isolated and cultured (36), and some of these common anaerobic fungi were detected in the present study. Similar to Griffith et al. (37) and Guo et al. (38), the results showed that *Orpinomyces* was the main anaerobic fungal genus in the rumen of yak. In addition to, *Cyllamyces, Anaeromyces*, and *Neocallimastix* were relatively high in abundance, but only the rhizoidal genus *Neocallimastix* was higher in abundance in CFG. This genus functions in the degradation of complex lignocellulosic plant biomass using highly active enzymes (39), but it can also could secrete amylases involved in the degradation of easily fermentable carbohydrates (40). Similar to this genus, other genera such as *Cyllamyces* can also rapidly colonize substrate surfaces of different carbon sources and thereby participate in the degradation of different diets (36). The results seemed to suggest that each genus had many functions and activities. Therefore, anaerobic fungi may widely participant in diet degradation in yak. In recent years, more and more results non-strictly anaerobic fungi have been identified in yak rumen samples (20, 38, 41). The non-strict anaerobic fungi *Ascomycota* and *Basidiomycota* were also found to be the dominant fungi in yak. This may be the result of a combination of environmental factors and host selection. However, most of these fungi came from the environment, and there has been no immediate concern about non-strict anaerobic fungi in the rumen of ruminants. Therefore, their metabolism and functions in the rumen ecosystem remain unclear.

Ruminant metabolites are needed for ruminant growth and health, and the proliferation of microorganisms is also closely related to metabolites. A change in feed type can alter the fermentation substrates of microorganisms, which in turn changes the metabolites in the rumen. VFAs are closely related to microbial fermentation and energy metabolism in the host. In this study, a high-fiber diet produced more acetate and high total VFA. One of the reasons may be the changes of bacteria (the genus *Ruminococcus 2* and *Bacteroidales BS11 gut group*) in the rumen mentioned in the previous article (21), and the other was the result of fungi changes. In this study, *Thermoascus* and *Cystofilobasidium* were more abundant in yaks fed the concentrate, in the meanwhile, the genera were negatively correlated with acetate and propionate concentrations. Previous study showed that *Thermoascus* had the ability to secrete highly active and thermostable CAZymes (42). And scientists have isolated a strain of the genus *Cystofilobasidium* capable of producing cold-active enzymes that degrade pectin (43). These suggested that *Thermoascus* and *Cystofilobasidium* were probably involved in carbohydrate metabolism. In addition, the concentration of branched-chain volatile fatty acids was also affected by the feed type. Branched-chain volatile fatty acids (valerate, isobutyrate, isovalerate) are important sources of carbon skeleton for microorganisms, are derived from branched-chain amino acids. The result showed the lower level of the isobutyrate and isovalerate concentrations in RFG. *Chrysosporium, Cladosporium* had the same changes as bacteria (the genus *Prevotellaceae UCG-003*), which were negatively correlated with branched volatile fatty acids. The inference was that the microbes in the CFG made more use of nutrient metabolites to meet their needs.

Previous studies have proved that feed type significantly affects protein digestion and absorption, and purine metabolism and other metabolic pathways in yak (21). In the present study, the results showed that correlations between different microorganisms and metabolites in the two groups were significantly different, indicating that rumen metabolism was closely related to feed type. Amino acids were among the metabolites significantly affected by feed type in the present study. In the rumen, amino acids mainly come from the degradation of dietary protein and microbial protein by the rumen microbial community. The higher concentration of NH_3_-N and amino acids in CFG indicated that more protein may be utilized and degraded. Fungi such as *Neocallimastix* possess proteolytic activity (44), and there are significant differences in protease activity between different anaerobic fungi (45). This may explain why *Neocallimastix* showed a strong positive correlation with individual amino acids. Meanwhile, protozoa cannot use ammonia and must ingest microbes to obtain certain nutrients (46). Similar to *Neocallimastix*, we found that *Thermoascus* and *Isotricha* were potentially related to protein metabolism, through degrading and/or ingesting for themselves. However, we still cannot ignore that bacteria play a major role in protein degradation. Fungi may form a consortium with bacteria that attaches to a feed particle, as well as protozoa regulates bacterial nitrogen turnover by gobbling up large molecules (47). Moreover, we observed that L-leucine, L-phenylalanine, L-tyrosine, and L-tryptophan were negatively associated with *Ascomycota*. Branched amino acids and aromatic amino acids are involved in many physiological activities. It has been reported that L-leucine can produce a large amount of isovalerate in the rumen, which can be further metabolized into acetoacetate and acetyl-CoA *via* the citric acid cycle as potential ketogenic substances (48). It is also an essential amino acid preferentially used by protozoa in the rumen (49). In addition, L-phenylalanine, L-tyrosine, and L-tryptophan are important glucogenic and ketogenic amino acids, and low levels may limit the growth and fermentation rate of rumen microorganisms (50). These Observation may have been the results of amino acids playing important roles in the growth and proliferation of microbe. Specifically, the amino acid β-alanine, which mainly forms carnosine and is not actually involved in protein formation, is a breakdown product of pyrimidine and normally metabolized into acetate (51). In the present study, β-alanine was negatively associated with the high abundance of certain genera in RFG. Therefore, it speculated that the degradation of β-alanine in the rumen of RFG yaks with lower starch and available carbohydrate levels may be one of the reasons for the higher acetate concentration.

Purine metabolism was also associated with the activity of some microbes in this study. Guanine and adenine are DNA and RNA bases that are catabolized to produce hypoxanthine and xanthine, and hypoxanthine can be further oxidized to xanthine, eventually producing uric acid. These molecules are combined with ribose or deoxyribose to form guanosine and adenosine. Adenosine, guanosine, guanine, adenine, xanthine, and hypoxanthine degradation products were detected in the present work. Similar to Ametaj et al. (52) and Zhang et al. (53), it found that the rumen environment of yaks fed concentrate may be more prone to microbial nucleic acid degradation. Furthermore, it was reported that anaerobic fungal genomes had an extremely high adenine-thymine (AT) content (54). Thus, we should pay more attention to the role of fungi in purine metabolism. Nowadays, most scientists use the purine derivative content of ruminant urine to calculate microbial protein production (17). However, in this study, changes in the concentrations of these metabolites in the rumen indirectly reflected the effects of diet on the internal environment.

The results of correlation analysis between oleic acid/palmitic acid and microbes indicated the effects of feed type on lipid metabolism. Palmitic acid is saturated, while oleic acid is monounsaturated, and it plays an important role in regulating the proliferation and differentiation of preadipocytes (55, 56). The composition of unsaturated fatty acids has an important positive effect on beef flavor (57), but they are rarely used in a free form in hosts. One of the reasons is that unsaturated fatty acids consumed from feed reach the rumen and are converted to saturated fatty acids by microbial hydrogenation, which are deposited in body fat. Previous studies showed that protozoa had no direct role in the hydrogenation of unsaturated fatty acids (58), but may be able to modulate the biohydrogenation. It reported that rumen protozoa can maintain pH by swallowing starch particles to avoid the negative transformation of biohydrogenated intermediates, as well as the unsaturated fatty acids combined with the protozoa membranes, avoiding the biohydrogenation of bacteria (59). In addition, rumen fungi catalyze biohydrogenation, but the hydrogenation activity was much lower than that of bacteria (60). Although the specific role of these fungi and protozoa in lipid metabolism in the rumen is still unclear, this correlation suggested that they may be involved in this process.

In addition, diet changes also affected the concentrations of other typical metabolites. D-maltose is a product of starch degradation by amylase. High-concentrate diets contain a large amount of easily fermentable carbohydrates, of which starch is the most important. This may explain the significant positive correlation between the D-maltose concentration and highly abundant genera in CFG. This also indicated that these fungi and protozoa may participate in the process of degrading unstructured carbohydrates. Niacin and choline are indispensable B vitamins in ruminants. Niacin acts as a direct precursor of important coenzymes NAD and NADP, and it is involved in the metabolism of nutrients in the body (61). It can be synthesized from tryptophan (62). The results showed that the change in L-tryptophan concentration was consistent with that of niacin. Thus, our study results revealed a strong association between affected microbiota and metabolites in important pathways; they not only showed the effects of feed type on rumen metabolism, but also that protozoa and fungi were closely related to metabolic processes. However, most microbiome eukaryotes remain poorly studied, hence we can't give precise conclusion.

## 5. Conclusion

In our study, feed with a high fiber content was more beneficial to protozoa growth and proliferation in the yak rumen, while fungi remained stable in high- and low-fiber diet groups. Feed type affected the microbiota, and altered some rumen metabolite concentrations. Both protozoa and fungi were found to be extensively involved in various metabolic processes, but fungi seem to be more active than protozoa in most processes. However, no individual rumen metabolite is independent of others, and the actions of multiple microbes may be important. This required further investigation. Regardless, this study contributes to a more comprehensive and complete understanding of rumen microbial composition and function, as well as important metabolites in yak. It provides more in-depth information on the yak rumen, and a reference for subsequent production and research. The findings also provide new avenues for exploration related to rumen microbes and rumen metabolism.

## Data availability statement

The datasets presented in this study can be found in online repositories. The name of the repository and accession number can be found at: SRA, NCBI; PRJNA665783.

## Ethics statement

The animal study was reviewed and approved by College of Animal Science and Technology, China Agriculture University, Beijing, P. R. China (permit number DK1402006).

## Author contributions

The study was designed by SL, SC, QM, and ZZ. Sample processing was carried out by HW. Data analysis was performed by XC. The manuscript was written by XC and modified by YL. All authors read and approved the final manuscript.

## References

[1] Weimer PJ. Redundancy, resilience, and host specificity of the ruminal microbiota: implications for engineering improved ruminal fermentations. Front Microbiol. (2015) 6:296. 10.3389/fmicb.2015.00296 25914693 PMC4392294

[2] Gordon GLR Phillips MW. Removal of anaerobic fungi from the rumen of sheep by chemical treatment and the effect on feed consumption and *in vivo* fibre digestion. Lett Appl Microbiol. (1993) 17:220–3. 10.1111/j.1472-765X.1993.tb01451.x

[3] Newbold CJ. de la Fuente G Belanche A Ramos-Morales E McEwan NR. The role of ciliate protozoa in the rumen. Front Microbiol. (2015) 6:1313. 10.3389/fmicb.2015.01313 26635774 PMC4659874

[4] Akin DE Borneman WS. Role of rumen fungi in fiber degradation. J Dairy Sci. (1990) 73:3023–32. 10.3168/jds.S0022-0302(90)78989-8 2178175

[5] Cheng Y Shi Q Sun R Liang D Li Y Li Y. The biotechnological potential of anaerobic fungi on fiber degradation and methane production. World J Microbiol Biotechnol. (2018) 34:155. 10.1007/s11274-018-2539-z 30276481

[6] Mackie RI Gilchrist FMC Robberts AM Hannah PE Schwartz HM. Microbiological and chemical changes in the rumen during the stepwise adaptation of sheep to high concentrate diets. J Agric Sci. (1978) 90:241–54. 10.1017/S0021859600055313

[7] Henderson G Cox F Ganesh S Jonker A Young W Janssen PH. Rumen microbial community composition varies with diet and host, but a core microbiome is found across a wide geographical range. Sci Rep. (2015) 5:14567. 10.1038/srep14567 26449758 PMC4598811

[8] Saleem F Bouatra S Guo AC Psychogios N Mandal R Dunn SM. The bovine ruminal fluid metabolome. Metabolomics. (2013) 9:360–78. 10.1007/s11306-012-0458-9

[9] Mao SY Huo WJ Zhu WY. Microbiome-metabolome analysis reveals unhealthy alterations in the composition and metabolism of ruminal microbiota with increasing dietary grain in a goat model. Environ Microbiol. (2016) 18:525–41. 10.1111/1462-2920.12724 25471302

[10] Zhang J Shi H Wang Y Li S Cao Z Ji S. Effect of dietary forage to concentrate ratios on dynamic profile changes and interactions of ruminal microbiota and metabolites in holstein heifers. Front Microbiol. (2017) 8:2206. 10.3389/fmicb.2017.02206 29170660 PMC5684179

[11] Qiu Q Zhang G Ma T Qian W Wang J Ye Z. The yak genome and adaptation to life at high altitude. Nat Genet. (2012) 44:946–9. 10.1038/ng.2343 22751099

[12] Xue D Chen H Luo X Guan J He Y Zhao X. Microbial diversity in the rumen, reticulum, omasum, and abomasum of yak on a rapid fattening regime in an agro-pastoral transition zone. J Microbiol. (2018) 56:734–43. 10.1007/s12275-018-8133-0 30136259

[13] Yang C Hou F Sun Y Yuan H Liu Y Zhang Y. Oats hay supplementation to yak grazing alpine meadow improves carbon return to the soil of grassland ecosystem on the Qinghai-Tibet Plateau, China. Global Ecol Conserv. (2020) 23:e01158. 10.1016/j.gecco.2020.e01158

[14] Huws SA Creevey CJ Oyama LB Mizrahi I Denman SE Popova M. Addressing global ruminant agricultural challenges through understanding the rumen microbiome: past, present, and future. Front Microbiol. (2018) 9:2161. 10.3389/fmicb.2018.02161 30319557 PMC6167468

[15] Xu T Xu S Hu L Zhao N Liu Z Ma L. Effect of dietary types on feed intakes, growth performance and economic benefit in tibetan sheep and yaks on the Qinghai-Tibet plateau during cold season. PLoS ONE. (2017) 12:e0169187. 10.1371/journal.pone.0169187 28056054 PMC5215856

[16] Ma L Xu S Liu H Xu T Hu L Zhao N. Yak rumen microbial diversity at different forage growth stages of an alpine meadow on the Qinghai-Tibet plateau. PeerJ. (2019) 7:e7645. 10.7717/peerj.7645 31579584 PMC6754979

[17] Zhou JW Liu H Zhong CL Degen AA Yang G Zhang Y. Apparent digestibility, rumen fermentation, digestive enzymes and urinary purine derivatives in Yaks and Qaidam Cattle offered forage-concentrate diets differing in nitrogen concentration. Livest Sci. (2018) 208:14–21. 10.1016/j.livsci.2017.11.020

[18] Zhang Z Xu D Wang L Hao J Wang J Zhou X. Convergent evolution of rumen microbiomes in high-altitude mammals. Curr Biol. (2016) 26:1873–9. 10.1016/j.cub.2016.05.012 27321997

[19] Fan Q Wanapat M Yan T Hou F. Altitude influences microbial diversity and herbage fermentation in the Rumen of Yaks. BMC Microbiol. (2020) 20:370. 10.1186/s12866-020-02054-5 33276718 PMC7718673

[20] Wu D Vinitchaikul P Deng M Zhang G Sun L Wang H. Exploration of the effects of altitude change on bacteria and fungi in the Rumen of Yak (Bos Grunniens). Arch Microbiol. (2021) 203:835–46. 10.1007/s00203-020-02072-x 33070234

[21] Liu C Wu H Liu S Chai S Meng Q Zhou Z. Dynamic alterations in Yak Rumen bacteria community and metabolome characteristics in response to feed type. Front Microbiol. (2019) 10:1116. 10.3389/fmicb.2019.01116 31191470 PMC6538947

[22] Teoh R Caro E Holman DB Joseph S Meale SJ Chaves AV. Effects of hardwood biochar on methane production, fermentation characteristics, and the Rumen microbiota using rumen simulation. Front Microbiol. (2019) 10:1534. 10.3389/fmicb.2019.01534 31354652 PMC6635593

[23] Chen J Bittinger K Charlson ES Hoffmann C Lewis J Wu GD. Associating microbiome composition with environmental covariates using generalized unifrac distances. Bioinformatics. (2012) 28:2106–13. 10.1093/bioinformatics/bts342 22711789 PMC3413390

[24] Paradis E. Schliep K. Ape 50: an environment for modern phylogenetics and evolutionary analyses in R. Bioinformatics. (2019) 35:526–8. 10.1093/bioinformatics/bty633 30016406

[25] Parks DH Tyson GW Hugenholtz P Beiko RG. Stamp: statistical analysis of taxonomic and functional profiles. Bioinformatics. (2014) 30:3123–4. 10.1093/bioinformatics/btu494 25061070 PMC4609014

[26] Guo W Zhou M Ma T Bi S Wang W Zhang Y. Survey of Rumen microbiota of domestic grazing yak during different growth stages revealed novel maturation patterns of four key microbial groups and their dynamic interactions. Anim Microbiome. (2020) 2:23. 10.1186/s42523-020-00042-8 33499950 PMC7807461

[27] dos Reis CC Maeda EM Cedrola F Martins EN De Paula FM Martinele I. Diet and breed alter community structures of rumen protozoa in cattle subjected to different feeding systems. Semina-Ciencias Agrarias. (2019) 40:909–18. 10.5433/1679-0359.2019v40n2p909

[28] Cedrola F Martinele I Dias RJ Fregulia P D'Agosto M. Rumen ciliates in brazilian sheep (Ovis Aries), with new records and redescription of entodinium contractum (Entodiniomorphida: Ophryoscolecidae). Zootaxa. (2016) 4088:292–300. 10.11646/zootaxa.4088.2.10 27394342

[29] Cedrola F Senra MVX Rossi MF Fregulia P D'Agosto M Dias RJP. Trichostomatid ciliates (alveolata, ciliophora, trichostomatia) systematics and diversity: past, present, and future. Front Microbiol. (2019) 10:2967. 10.3389/fmicb.2019.02967 32010077 PMC6974537

[30] Coleman GS. The rate of uptake and metabolism of starch grains and cellulose particles by Entodinium species, Eudiplodinium maggii, some other entodiniomorphid protozoa and natural protozoal populations taken from the ovine rumen. J Appl Bacteriol. (1992) 73:507–13. 10.1111/j.1365-2672.1992.tb05013.x 1490912

[31] Huws SA Kim EJ Kingston-Smith AH Lee MR Muetzel SM Cookson AR. Rumen protozoa are rich in polyunsaturated fatty acids due to the ingestion of chloroplasts. FEMS Microbiol Ecol. (2009) 69:461–71. 10.1111/j.1574-6941.2009.00717.x 19583786

[32] Huws SA Lee MRF Kingston-Smith AH Kim EJ Scott MB Tweed JKS. Ruminal protozoal contribution to the duodenal flow of fatty acids following feeding of steers on forages differing in chloroplast content. Br J Nutr. (2012) 108:2207–14. 10.1017/S0007114512000335 22377337

[33] Firkins JL Yu Z Park T Plank JE. Extending burk dehority's perspectives on the role of ciliate protozoa in the Rumen. Front Microbiol. (2020) 11:123. 10.3389/fmicb.2020.00123 32184759 PMC7058926

[34] Ho YW Abdullah N. The role of rumen fungi in fibre digestion - review. Asian-australas. J Anim Sci. (1999) 12:104–12. 10.5713/ajas.1999.104

[35] Gruninger RJ Puniya AK Callaghan TM Edwards JE Youssef N Dagar SS. Anaerobic fungi (Phylum Neocallimastigomycota): advances in understanding their taxonomy, life cycle, ecology, role and biotechnological potential. FEMS Microbiol Ecol. (2014) 90:1–17. 10.1111/1574-6941.12383 25046344

[36] Wang X Benoit I Groenewald JZ Houbraken J Dai X Peng M. Community dynamics of neocallimastigomycetes in the rumen of yak feeding on wheat straw revealed by different primer sets. Fungal Ecol. (2019) 41:34–44. 10.1016/j.funeco.2019.03.007

[37] Griffith GW Ozkose E Theodorou MK Davies DR. Diversity of anaerobic fungal populations in cattle revealed by selective enrichment culture using different carbon sources. Fungal Ecol. (2009) 2:87–97. 10.1016/j.funeco.2009.01.005

[38] Guo W Wang W Bi S Long R Ullah F Shafiq M. Characterization of anaerobic rumen fungal community composition in yak, tibetan sheep and small tail han sheep grazing on the Qinghai-Tibetan plateau. Animals. (2020) 10:144. 10.3390/ani10010144 31963125 PMC7023293

[39] Solomon KV Haitjema CH Henske JK Gilmore SP Borges-Rivera D Lipzen A. Early-branching gut fungi possess a large, comprehensive array of biomass-degrading enzymes. Science. (2016) 351:1192–5. 10.1126/science.aad1431 26912365 PMC5098331

[40] Kelleci BM Comlekcioglu U. Production of amylolytic enzyme by rumen fungi, neocallimastix Sp K7 and orpinomyces Sp K5. J Anim Plant Sci. (2016) 26:242–52.

[41] Kumar S Indugu N Vecchiarelli B Pitta DW. Associative patterns among anaerobic fungi, methanogenic archaea, and bacterial communities in response to changes in diet and age in the rumen of dairy cows. Front Microbiol. (2015) 6:781. 10.3389/fmicb.2015.00781 26284058 PMC4521595

[42] Gabriel R Mueller R Floerl L Hopson C Harth S Schuerg T. Cazymes from the thermophilic fungus thermoascus aurantiacus are induced by C5 and C6 Sugars. Biotechnol Biofuels. (2021) 14:169. 10.1186/s13068-021-02018-5 34384463 PMC8359064

[43] Tomoyuki N Kaichiro Y Tatsuro M Noboru T. Cold-active pectinolytic activity of psychrophilic-basidiomycetous yeast. J Biosci Bioeng. (2002) 94:175–7. 10.1016/S1389-1723(02)80140-2 16233289

[44] Asao N Ushida K Kojima Y. Proteolytic activity of rumen fungi belong to the genera neocallimastix and piromyces. Lett Appl Microbiol. (1993) 16:247–50. 10.1111/j.1472-765X.1993.tb01410.x

[45] Michel V Fonty G Millet L Bonnemoy F Gouet P. *In vitro* study of the proteolytic activity of rumen anaerobic fungi. FEMS Microbiol Lett. (1993) 110:5–10. 10.1111/j.1574-6968.1993.tb06287.x 8319894

[46] Belanche A de la Fuente G Moorby JM Newbold CJ. Bacterial protein degradation by different rumen protozoal groups. J Anim Sci. (2012) 90:4495–504. 10.2527/jas.2012-5118 22829613

[47] Bach A Calsamiglia S Stern MD. Nitrogen metabolism in the Rumen. J Dairy Sci. (2005) 88:E9–E21. 10.3168/jds.S0022-0302(05)73133-7 15876575

[48] Menahan LA Schultz LH. Metabolism of Leucine + Valine within Rumen. J Dairy Sci. (1964) 47:1080. 10.3168/jds.S0022-0302(64)88849-4

[49] Ahuja SP. Sarmah TC. Studies on the activity of rumen protozoa II utilization of U-C-14-L-Leucine and U-C-14-L-Serine by Rumen protozoa zentralblatt fur veterinarmedizin reihe a-journal of veterinary medicine series a-animal physiology. Pathol Clin Vet Med. (1979) 26:551–7. 10.1111/j.1439-0442.1979.tb01631.x 116449

[50] Guliye AY Wallace RJ. Effects of aromatic amino acids, phenylacetate and phenylpropionate on fermentation of xylan by the rumen anaerobic fungi, neocallimastix frontalis and piromyces communis. J Appl Microbiol. (2007) 103:924–9. 10.1111/j.1365-2672.2007.03327.x 17897195

[51] Schnuck JK Sunderland KL Kuennen MR Vaughan RA. Characterization of the metabolic effect of beta-alanine on markers of oxidative metabolism and mitochondrial biogenesis in skeletal muscle. J Exerc Nutr Biochem. (2016) 20:34–41. 10.20463/jenb.2016.06.20.2.5 27508152 PMC4977905

[52] Ametaj BN Zebeli Q Saleem F Psychogios N Lewis MJ Dunn SM. Metabolomics reveals unhealthy alterations in rumen metabolism with increased proportion of cereal grain in the diet of dairy cows. Metabolomics. (2010) 6:583–94. 10.1007/s11306-010-0227-6

[53] Zhang R Zhu W Jiang L Mao S. Comparative metabolome analysis of ruminal changes in holstein dairy cows fed low- or high-concentrate diets. Metabolomics. (2017) 13:74. 10.1007/s11306-017-1204-0

[54] Brownlee AG. Remarkably at-rich genomic DNA from the anaerobic fungus neocallimastix. Nucleic Acids Res. (1989) 17:1327–35. 10.1093/nar/17.4.1327 2922283 PMC331806

[55] Liao FH Liou TH Shieh MJ Chien YW. Effects of different ratios of monounsaturated and polyunsaturated fatty acids to saturated fatty acids on regulating body fat deposition in hamsters. Nutrition. (2010) 26:811–7. 10.1016/j.nut.2009.09.009 20022469

[56] Ferlay A Bernard L Meynadier A Malpuech-Brugere C. Production of trans and conjugated fatty acids in dairy ruminants and their putative effects on human health: a review. Biochimie. (2017) 141:107–20. 10.1016/j.biochi.2017.08.006 28804001

[57] Lee HJ Lee SC Oh YG Kim YH Kim HB Park YH. Effects of rumen protected oleic acid in the diet on animal performances, carcass quality and fatty acid composition of hanwoo steers. Asian-australas J Anim Sci. (2003) 16:1003–10. 10.5713/ajas.2003.1003

[58] Devillard E McIntosh F Newbold C Wallace R. Rumen ciliate protozoa contain high concentrations of conjugated linoleic acids and vaccenic acid, yet do not hydrogenate linoleic acid or desaturate stearic acid. Br J Nutr. (2006) 96:697–704. 10.1079/BJN20061884 17010229

[59] Francisco AE Santos-Silva JM V. Portugal AP Alves SP B. Bessa RJ. Relationship between rumen ciliate protozoa and biohydrogenation fatty acid profile in rumen and meat of lambs. PLoS ONE. (2019) 14:e0221996. 10.1371/journal.pone.0221996 31490993 PMC6730912

[60] Nam IS Garnsworthy PC. Biohydrogenation of linoleic acid by rumen fungi compared with rumen bacteria. J Appl Microbiol. (2007) 103:551–6. 10.1111/j.1365-2672.2007.03317.x 17714387

[61] Gao Z Li Y Xu C Luo D Qiu Q Pan K. Niacin mitigates rumen epithelial damage *in vivo* by inhibiting rumen epithelial cell apoptosis on a high concentrate diet. Vet Res Commun. (2022) 46:699–709. 10.21203/rs.3.rs-884917/v1 35076856

[62] Niehoff ID Huther L Lebzien P. Niacin for dairy cattle: a review. Br J Nutr. (2009) 101:5–19. 10.1017/S0007114508043377 18702847

